# Within-host mathematical modelling of the incubation period of *Salmonella* Typhi

**DOI:** 10.1098/rsos.182143

**Published:** 2019-09-11

**Authors:** Adedoyin Awofisayo-Okuyelu, Adrian Pratt, Noel McCarthy, Ian Hall

**Affiliations:** 1 National Institute of Health Research Health Protection Research Unit in Gastrointestinal Infections, University of Oxford, Oxford, UK; 2 Department of Zoology, University of Oxford, Oxford, UK; 3 Emergency Response Department Science and Technology (ERD S&T), Health Protection Directorate, Public Health England, Porton Down, UK; 4 Warwick Medical School, University of Warwick, Coventry, UK; 5 School of Mathematics, University of Manchester, Manchester, UK

**Keywords:** incubation period, *Salmonella* Typhi, mathematical modelling

## Abstract

Mechanistic mathematical models are often employed to understand the dynamics of infectious diseases within a population or within a host. They provide estimates that may not be otherwise available. We have developed a within-host mathematical model in order to understand how the pathophysiology of *Salmonella* Typhi contributes to its incubation period. The model describes the process of infection from ingestion to the onset of clinical illness using a set of ordinary differential equations. The model was parametrized using estimated values from human and mouse experimental studies and the incubation period was estimated as 9.6 days. A sensitivity analysis was also conducted to identify the parameters that most affect the derived incubation period. The migration of bacteria to the caecal lymph node was observed as a major bottle neck for infection. The sensitivity analysis indicated the growth rate of bacteria in late phase systemic infection and the net population of bacteria in the colon as parameters that most influence the incubation period. We have shown in this study how mathematical models aid in the understanding of biological processes and can be used in estimating parameters of infectious diseases.

## 1. Background

Typhoid fever is a systemic infection caused by the bacteria *Salmonella* Typhi. It is endemic in developing countries; however, a sporadic disease in developed countries with infection occurring mainly in travellers returning from endemic regions [1]. Community-wide outbreaks associated with widely distributed products [2,3] or water supply [4–6] are commonly reported as well as point source outbreaks associated with dairy [7], red meat [8,9] and vegetables [10,11] have been reported.

Possible control measures that can be applied to typhoid fever depend on the population. In developing countries, this will involve provision of potable drinking water and hygienic food preparation [1]. In developed countries, activities such as prompt investigation of outbreaks, identification of travel-related cases and confirming cases as either sporadic or outbreak-related, all of which require accurate knowledge of the incubation period, contribute to reducing the burden of disease. Furthermore, the efficacy of typhoid vaccine has been tested in experimental and field studies.

The incubation period of typhoid fever is defined as the time between the exposure to the pathogen and the onset of clinical illness. Individual variation in the incubation period, due to host and pathogen characteristics, means that this parameter is reported as a distribution rather than a single estimate. In addition to understanding the pathophysiology of the disease, accurate knowledge of the distribution of incubation period is important for surveillance, outbreak investigations and conducting epidemiological and ecological studies [12]. Available reports on the incubation period of *S*. Typhi vary extensively. Some community-wide outbreaks have reported incubation periods with a mean of 19 days [13] or a range of 2–11 days [14] and some point source outbreaks have reported ranges of 5–12 days [7] and 1–28 days [15].

International health organizations, such as WHO and CDC [16,17], report the incubation period as ranging from 3 to 60 days and 3 to 30 days, respectively, possibly in an attempt to include the possible extremes of the distribution. These estimates are probably based on the observations from a limited number of studies and factors influencing the distribution are unknown. Understanding the distribution of incubation period and the influencing factors may be possible by undertaking a systematic review of observational or experimental studies; however, mathematical models have the potential to provide a more complete description and identify influencing factors not detected using other research methods.

Mathematical models have been employed in understanding areas of infectious diseases such as the spread of a pathogen within a host [18] and the immune process of the human body [19]. They can also be used to estimate parameters like incubation period and dose–response or predict outcomes by fitting data to the developed models. The process of model formulation helps us to increase the understanding of the pathophysiology, clarify and document assumptions and data gaps. It is also useful in assessing the sensitivity of the model output to changes in parameter values in an attempt to identify influencing factors.

We developed a compartmental model that described the process of infection from ingestion of a contaminated liquid meal to the onset of clinical illness using a set of ordinary differential equations. Each equation represented the transition from one compartment to the next and estimated the duration of each step. The model was parametrized using estimated values identified from the literature. Observed values from the model output were compared to expected values identified from the data of a human experimental study. We also conducted a sensitivity analysis to identify parameters or variables that influence the distribution of the incubation period. Our model represents infection in a naive case or population which has not been previously exposed to or infected with *S*. Typhi.

## 2. Methods

### 2.1. Model formulation

**Figure 1.**
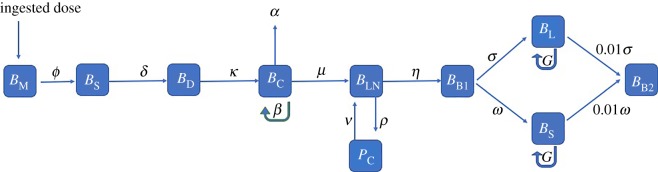
Flowchart of the model showing the mathematical representation of the infection process.

We summarized the pathophysiology of *S*. Typhi into mathematical equations and developed a compartmental model. This represented the transfer of bacteria per unit time from initial ingestion to the onset of clinical symptoms (mouth to the secondary bacteraemia) (figure 1). Ordinary differential equations were used to describe the rate of change of bacteria in each compartment which included a combination of the processes: migration, replication or clearance (electronic supplementary material, appendix S1). The model was solved using the deSolve package in the R statistical software [20].

### 2.2. Model parametrization

The model has 17 parameters (we do not include the initial number of bacteria ingested as a parameter) and parameter values were derived from the literature. Reviewing the available evidence for the various transit times and rates, we identified, calculated and estimated values for our model parameters. Where possible, values indicating duration or proportion were converted to rates. Bacteria transit times in the gastrointestinal tract and flow rates in the lymph and blood were estimated using the identified human experimental studies. At cellular level, we assumed that the replication and death of bacteria and their interactions with macrophages are similar in mice and humans, hence, we used several mouse models of *Salmonella* typhimurium (*S*. typhimurium) infection to describe cellular infection in humans [18,21,22].

### 2.3. Uncertainty sampling and sensitivity analyses

The parameter values inputted in our model have some degree of uncertainty due to factors such as natural variation between individuals or measurement error in experiments. We, therefore, conducted an uncertainty analysis to quantify the degree of uncertainty in the parameter estimates and then a sensitivity analysis to explore the effect of changes in the model parameters on the incubation period. The sensitivity analysis was conducted in a two-step process: first, determining the effect of each parameter individually on the incubation period and identifying the most sensitive parameters; then determining the combined effect of all identified sensitive parameters on the incubation period. We used a computational modelling software called Sampling and Sensitivity Analysis Tool (SaSAT) developed by Hoare *et al.* [23] for both the uncertainty sampling and the sensitivity analysis.

#### 2.3.1. Uncertainty sampling

In conducting the uncertainty analysis, we employed Latin hypercube sampling (LHS) method, a form of Monte–Carlo simulation. It uses stratified sampling [24] to randomly select parameter values within the given parameter distributions that result in an unbiased estimate of the average model output [25]. For each parameter value, we specified a range from which the random values were selected (table 1). Some of the parameter values had corresponding measures of variation such as standard deviation or 95% confidence interval, hence the range specified was based on empirical evidence. For other parameter values, the range was selected based on the best available evidence from the literature or best-guess estimates-based biological expectations. We also defined the underlying distribution of each parameter value from which the random samples were selected. A triangular distribution was mostly chosen in the absence of evidence for other type of distribution, and this included a peak value and plausible minimum and maximum values. Due to the triangular shape of the distribution, more random samples were selected around the peak value. A uniform distribution was selected when there was neither evidence of other type of distribution, nor evidence supporting the parameter value. In this case, minimum and maximum values are selected to give a range, which could include the actual parameter value. The uniform sampling from this range, due to the uncertainty of the parameter value, produces a uniform distribution. The parameter estimates, range and underlying distribution, used to derive the random parameter values, are summarized in table 1.

**Table 1.** Values, range and distribution of parameter values used in the sensitivity analysis.

parameter	description	parameter value	unit	range	reference	distribution	rationale for values, range and distribution
*B*_0_	ingested dose		counts				
*ϕ*	transit rate through the mouth		rate per hour				
*δ*	gastric-emptying rate	1.4	rate per hour	s.d. ± 0.7	Bennink *et al.* [26]	normal	multiple published evidence available. Data chosen as authors reported overall mean (±s.d.) emptying rate for liquids
*γ*	fraction of bacteria entering the small intestine (SI)	0.95	proportion	range: 0.8–1		uniform	value is assumed to be 95% as the literature indicates almost all bacteria enter the SI. The values of the range were chosen to include an upper value that allows for 100% of bacteria to go through considering the food vehicle of the experimental study
*κ*	small intestine emptying rate	0.3	rate per hour	s.d.± 0.1	Yu & Amidon [27]	normal	multiple published evidence available. Value chosen as data were estimated from over 400 human transit data. Evidence suggests that transit time distribution is not normal or lognormal
*α*	rate of bacteria shedding from the colon	0.36	rate per hour	range: 0.1–1.9		uniform	value was selected based on best-guess estimate. Expecting that some proportion of bacteria must be shed from the colon, the lower limit of the range was set at 0.1. The upper limit was set to be below the replication rate, because if shedding occurs faster than replication, infection will be cleared
*β*	replication rate in the colon	0.43	rate per hour	range: 0.1–2.1	Knodler *et al.* [28]	triangular	value based on published evidence. Parameter value is the average doubling time. Upper limit represents the replicating rate of the hyper-replicating cells reported by Knodler *et al*. Lower limit is a best-guess estimate of the slowest possible replication rate. As no distribution is available in the literature, a triangular distribution is selected to include the min and max values
*μ*	transfer rate to the caecal lymph node	1.25 × 10^−8^	rate per hour	range: 0–5.5 × 10^−8^	Kaiser *et al.* [29]	triangular	published evidence reports mean value and 95% CI. The corresponding s.e.m. from the 95% CI was used to derive the s.d. in order to extend the range around the mean. A triangular distribution was selected to include the mean and +1.96 × s.d. as the upper limit of the range and lower limit was censored at 0 as a negative migration rate would result in negative model outputs
*θ*	fraction of bacteria replicating in a phagocyte	0.3	proportion	range: 0.1–0.6		triangular	parameter value is the average number reported across three experiments. From the range of values reported in the experiments, the lower and upper limit of the range were selected to be minimum and maximum values reported in the experiments, expecting that the fraction of bacteria available to replicate cannot be less than 10% or more than 60%
*ρ*	rate of phagocyte invasion	0.09	rate per second	range: 0.018–0.162	Gog *et al.* [30]	triangular	two values were reported in the literature from a physical model and a mathematical model. The value from the physical model was selected as the more biologically plausible of the two as it did not depend on the multiplicity of infection (MOI), which is difficult to ascertain during infection. The upper limit of the range was selected to be similar to the value reported in the mathematical model as the maximum possible rate
*ν*	rate of phagocyte rupture	0.41	rate per hour	range: 0.20–0.60	Monack *et al.* [31]	triangular	the values of the range were selected based on best-guess estimates. If the rupture rate is below 0.1 or close to 1, infection might not occur. If the phagocytes rupture too slowly, there will be more bacterial death occurring; and if the phagocyte rupture to quickly, there will be insufficient replication of bacteria within the phagocyte
*C*	number of bacteria in phagocyte at rupture	4.1	counts	range: 1–10	calculated	triangular	the range of values selected would allow for a lower but longer tail, while still centred around the peak value
*η*	flow rate from lymph to blood	0.025	rate per hour	range: 0.005– 0.315	Frietas [32]	triangular	parameter estimate is selected from the report of Frietas. The upper limit of the range is the value reported by Alexander *et al.* [33] which would represent the fastest flow rate
*σ*	mean blood flow rate in portal vein	7.9	rate per hour	s.d. ± 2	Brown *et al.* [34]	normal	values based on published evidence. It is assumed that the flow rate follows a normal distribution with the reported standard deviation around the mean
*ω*	mean blood flow rate in the splenic artery	2.4	rate per hour	s.d. ± 0.4	Sato *et al.* [35]	normal	values based on published evidence. It is assumed that the flow rate follows a normal distribution with the reported standard deviation around the mean
*τ* (represented by *τ*_1_ and *τ*_2_)	net growth rate in the early and late stages of systemic colonization	−1.04 in the early phase 0.09 in the late phase	rate/hour	range in the early phase (*τ*_1_): −2 to 0 range in the late phase (*τ*_2_): 0–1	Grant *et al.* [18]	triangular	in the early phase, bacterial death is higher than replication. The range is set so that at the upper limit, death rate is equal to replication rate, and at the lower limit, death rate is twice the replication rate. in the late phase, bacterial death is negligible; thus, the range is set such that at the lower limit, replication rate is equally negligible and at the upper limit, the replication rate is one magnitude higher
Υ	magnitude of reduction in flow rate from systemic organs back to blood	0.01	proportion	0.001 to 0.1	Grant *et al.* [18]	triangular	best-guess estimate that proportional change in flow rate can be as low as 0.1% to and no higher than 10%

#### 2.3.2. Sensitivity analysis

In order to examine the sensitivity of each parameter independently, the single-parameter models were run with the values of the parameter of interest varying according to the samples generated by the LHS, while the values of the other parameters were fixed. For example, when examining the sensitivity of *δ*, the model was run with varying values of *δ* and fixed values for the other parameter. Using the 100 random samples generated by the LHS, 100 reiterations were run for each parameter considered in the sensitivity resulting in 16 sets of 100 model outputs (we did not include the transit time from mouth to stomach in the sensitivity as this was such a short duration).

## 3. Results

Parameter values were derived to solve the ordinary differential equations. A list of the estimated parameter values, to be included in the model, is available in table 1.

### 3.1. Model parametrization

#### 3.1.1. Gastric-emptying rate (*δ*)

Following ingestion, the bacteria transits through the mouth and arrives in the stomach within a few seconds (*ϕ*) (this parameter is not tested for sensitivity as it is so short a duration).

According to Bennink *et al.* [26], the liquid-emptying rates for both males and females are similar (2.34% min^−1^ and 2.33% min^−1^, respectively), hence, we decided on a single emptying rate for both genders of 2.3% min^−1^. We calculated the average emptying time using the equation

3.1a $$p(t)=\;1\;-e^{-\delta t},$$

where *p*(*t*) is the proportion emptied per minute (0.023) and *t* is time (1 min), therefore

3.1b $$\begin{array}{rl} \delta = & \;\frac{\ln(1-p(t))}{t}\\ \delta = & \frac{-1}{\ln(1-0.023)}=42.9. \end{array}\}$$

It will take an average of 43 min for gastric content to be emptied. Calculating the reciprocal of the emptying time results in a rate of 1.40 h^−1^ (1/(43/60)).

#### 3.1.2. Fraction of bacteria migrated to duodenum (γ)

For the purpose of our model, we assumed that gastric acid was neutralized and the altered gastric pH increases the likelihood of the ingested bacteria to pass through the stomach unrestricted. Thus, we estimated that about 95% (0.95) of the ingested bacteria migrated to the duodenum.

#### 3.1.3. Small bowel transit time (*κ*)

The compartmental transit model, developed by Yu & Amidon [27], can be used to characterize the variation in the population. The model assumes that the gastric content flows through the small intestine by passing through a series of segments/compartments, where each compartment has different food volumes and flow rates but equal transit times. The mean transit time can be derived, and the ideal number of compartment (*N*) can be determined from the compartmental analysis by adding and subtracting compartments until the residual sum of squares (SSE) becomes small. From their analysis, Yu *et al.* [36] identified a seven-compartment transit model as the best model to describe the flow process.

Reproducing the analysis from Yu *et al.*, the values of *N* with the smallest SSE were 6.9 and 7.0 and the corresponding SSE were 10.8 and 11.1, respectively. Although the SSE for 6.9 was the smallest, inputting non-integers into our model could not be justified as it is not possible to have a fraction of a model compartment. Hence, we opted for seven compartments and a mean transit time of 199 min (*κ*), which was the same value reported by Yu *et al.* [36]. This resulted in an emptying rate of 0.30 h^−1^.

#### 3.1.4. Bacteria in the colon (*β*, *μ* and *α*)

The average doubling time of *Salmonella* in the colon, according to Knodler *et al.* [28], was 95 min. From the doubling time formula

3.2 log2=rt,

where *r* and *t* are the replication rate and doubling time (in hours), respectively; we solved for *r* as r=log2/t. The resulting replication rate was *β* = 0.43.

According to the combined experimental study and mathematical models undertaken by Kaiser *et al.* [29], the resulting probability of migration in the first 24 h was 3 × 10^−7^. Therefore, the probability of a bacterium migrating in 1 h is *μ* = 1.25 × 10^−8^.

Considering the high replication rate of bacteria in the colon and the low probability of migrating to the caecal lymph node, more bacteria will be shed through faeces compared to the quantity that continue towards systemic infection.

However, for gut invasion to be sustained and for infection to progress, the replication rate of bacteria in the colon should be higher than the rate it sheds through faeces. In order to avoid total bacterial clearance, the net bacterial population needs to be greater than 1. Considering that the average doubling time of bacteria is approximately 95 min, we would expect that for gut invasion to be sustained, the clearance of bacteria would occur at a slower rate and the time it takes bacteria to shed from the colon would be nearly twice as long as the doubling time. In the absence of evidence in the literature, a shedding time that is 1.8 times the doubling time is our best-guess estimate, allowing the net population of bacteria in the colon to be greater than 1, yet not resulting in a very slow rate that may unreasonably prolong the incubation period. This results in a shedding time of 171 min and a corresponding shedding rate per unit time of 0.35 h^−1^.

#### 3.1.5. Rate of phagocyte invasion/engulfment (*ρ*)

Based on the physical model conducted by Gog *et al.* [30], the rate of phagocyte invasion was 2.5 × 10^−5^ s^−1^ per bacterium or 0.09 h^−1^.

#### 3.1.6. Proportion of bacteria replicating in a phagocyte (*θ*)

Van Dissel *et al.* [37] carried out experiments and developed a model to estimate the intracellular killing of bacteria using phagocytes from three types of mice. In both the experiments and the model, the number of viable bacteria in the phagocyte decreased after 90 min in all three types of macrophages. In the experiment, the percentage of viable bacteria decreased to 69, 25 and 13%, while from the developed model, the number of bacteria decreased to about 20% in two types of macrophages and to 50% in the third [37].

Based on the experiment and model developed by van Dissel *et al.* [37], the average proportion of viable bacteria available in the cell after phagocytosis in the three types of mice was approximately 30%.

#### 3.1.7. Growth rate of bacteria in phagocyte

Extracting the experimental data from Forest *et al.* [38] for the increase in intracellular bacterial population, we derived the replication rate of intracellular bacteria. We fitted the extracted data to a logistic replication model (electronic supplementary material, appendix S2)

3.3 $$P=\frac{CB_{0}}{B_{0}+(C-B_{0})e^{-rt}},$$

where *P* is the number of bacteria in the phagocyte after replication, *B*_0_ is the initial number of bacteria in the phagocyte and *C*, *r*, and *t* represent the maximum value (the ‘carrying capacity’), exponential growth and time, respectively. The predicted intracellular growth rate was 0.3 h^−1^ in the first 6 h after infection which results in a doubling time of 2.3 h.

#### 3.1.8. Rate of phagocyte rupture (*ν*)

Data from Monack *et al.* [31], on the proportion of dead phagocytes at different time intervals after infection, were extracted. The median rupture time, which is the time it will take for 50% of the phagocytes to rupture, is chosen to be representative of the average rupture time. We fitted the data to a logistic model (after checking the fit against one using a lognormal distribution)

3.4 $$f(x)=\frac{1}{1+e^{-k(x-x_{0})}}.$$

The resulting intercept and gradient were 4.59 and −0.03179, respectively. Inputting these parameters into equation (3.4) and solving for *x* resulted in a value of 144.38 (4.59/−(−0.03179)). Coincidentally, this value also represented the ‘median rupture time’; hence, the time it took for 50% of phagocytes to rupture was 144.38 min or 2.41 h. This corresponds to a rupture rate of 0.41 h^−1^.

#### 3.1.9. Population of bacteria in phagocyte at rupture (*C*)

The number of bacteria in the phagocyte at the time of rupture depends on the rate of intracellular bacteria replication and rate of phagocyte rupture. Using the replication rate predicted from the model in equation (3.3), and the median rupture time from the model in equation (3.4), we derived the number of bacteria in the phagocyte at the time of rupture.

According to an experiment by Mitsuhashi *et al.* [39], when phagocyte cells and bacteria were incubated at a ratio of 1–10, the proportion of infected phagocyte cells was 30% of which 80% ingested one to two bacteria. We then assumed that at the time of phagocytosis, each cell would contain a maximum of two bacteria. Inputting the derived doubling time of 2.3 h and the required duration of 2.4 h, which is the median rupture time, into the formula *B*(2)*t*/*d*, where *B* is the initial number of bacteria available, *t* is the period of interest and *d* is the doubling time, we can estimate that the number of bacteria in the phagocyte at rupture would be 4.1.

#### 3.1.10. Draining rate of lymph from the caecal lymph node (*η*)

In order to estimate the flow rate out of the caecal lymph node, we assumed that bacteria drain from the caecal lymph node at the same rate that lymph flows through the intestinal lymphatic vessel. The vessel has a flow velocity of 3400 µm s^−1^ and a corresponding flow rate of 14 mm^3^ s^−1^ [32]. Equivalent to 0.014 ml s^−1^. The lymph vessels in the human body have a total capacity of approximately 2 l [32]; therefore, the intestinal lymphatic vessel rate is 0.025 h^−1^.

#### 3.1.11. Transfer rate into systemic organs (*σ* and *ω*)

In order to estimate the transfer rate of bacteria into the organs, we assumed that bacteria will travel into organs at the same rate as blood flow in the portal vein and splenic arteries and that equal proportion of bacteria enter into both organs. The liver receives about 70% of its blood supply via the portal vein, which is also the direct venous outflow from the intestine [40]. In the light of this, we can conclude that bacteria would enter into the liver via the portal vein. The mean blood velocity in the portal vein was reported to be 13.9 ± 4.49 cm s^−1^ and the resulting mean volume flow was 662 ± 169 ml min^−1^ [33]. Considering that the total blood volume of a typical adult is 5 l (5000 ml), the transfer rate into the portal vein is, therefore, 0.13 min^−1^.

According to Sato *et al.* [35], the mean blood velocity in the splenic artery was 18.7 ± 4.2 cm s^−1^ and the resulting mean blood flow rate was 179 ± 37 ml min^−1^. The transfer rate into the spleen was 0.04 min^−1^.

#### 3.1.12. Net growth of bacteria in systemic organs (*τ*)

According to the mouse model and accompanying mathematical model developed by Grant *et al.* [18], the bacterial population in the organs slowly decreases in the first 6 h with a doubling time of 1.7 h and a half-life of 1.1 h. The replication/birth rate was calculated using the formula in equation (3.2), and the death rate was calculated using the half-life formula

3.5 log0.5=rt.

The resulting birth rate and death rate were 0.41 and −0.63, respectively. Thus, the net growth rate for both the spleen and liver was −1.04 in the first 7 h post primary bacteraemia during the invasion phase.

In the replication phase of exponential bacterial growth, the doubling time decreases to 8 h [18] resulting in a net growth rate of 0.09.

#### 3.1.13. Transfer rate of bacteria from systemic organs into the blood and onset of the secondary bacteraemia

Based on the mouse model by Grant *et al.* [18], the relative rate of bacterial transfer out of the systemic organs was about 1% of the bacterial transfer rate from blood to the organs (σ and *ω*). Therefore, the transfer rates from the liver and spleen are 0.0013 min^−1^ and 0.0004 min^−1^, respectively.

### 3.2. Model analysis

From our model, we observed three critical stages for infection mathematically, and perhaps biologically, including: the net population of bacteria in the colon (determined by *β* > *α*), the bacterial population during phagocytosis (determined by *θC* > 1) and the growth rate in late systemic infection (determined by *τ*_2_ > 0). Most compartments in the model act to transit the bacterial load to other locations in the body or remove bacteria. If these three conditions on parameters are not met, then bacterial population will reduce over time and result in no infection.

### 3.3. Solving the model and interpretation

The initial state of each compartment was defined. We defined *B*_0_ as the initially ingested dose of bacteria with a concentration of 10^4^. We, therefore, set initial conditions such that all states are zero except *B_M_*(0) = *B*_0_.

**Figure 2.**
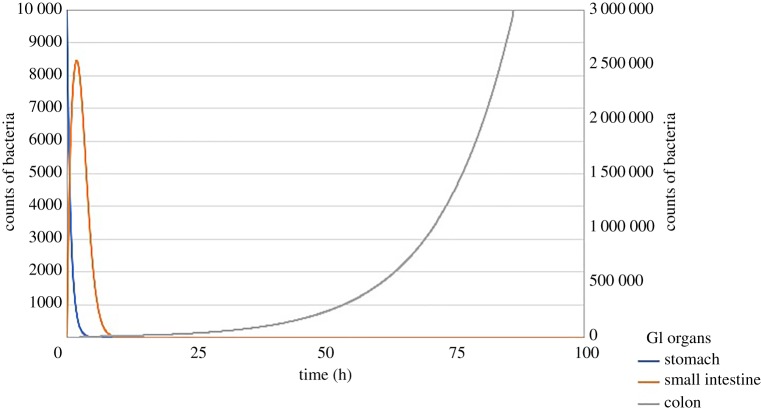
Model showing the initial gastrointestinal (GI) phase of the infection process.

The transfer of bacteria through the stomach, small intestine and colon is represented in figure 2. Gastric contents are completely emptied within 5 h of ingestion. Transit through the small intestine follows a gamma distribution and using the seven subcompartments of the small intestine [36] in the model resulted in a slight delay before the concentration of bacteria in the colon begins to increase (figure 2).

**Figure 3.**
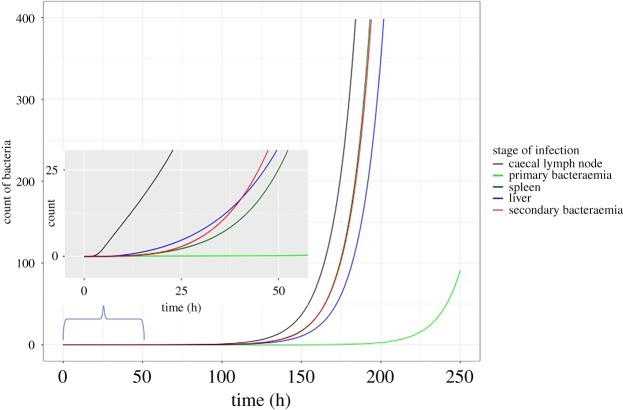
Model showing invasion of caecal lymph node to the onset of the secondary bacteraemia.

The invasion of the caecal lymph node to the onset of the secondary bacteraemia is represented in figure 3. At the early stages of systemic infection, the concentration of bacteria in the liver was higher than that in the spleen and blood, but as the infection progressed, bacterial concentration in the spleen and blood increased.

### 3.4. Estimating incubation period

Using the experimental study conducted by Waddington *et al.* [41], we estimated the incubation period from our model output. In the experimental study, participants were challenged with a dose of 10^4^ and monitored until typhoid diagnosis (TD). The median incubation time to TD was 8 days (IQR 6–9). Quantitative blood culture performed at the time of TD showed median bacterial loads of 1.1 CFU ml^−1^ (IQR 0.4–2.1).

From the mathematical model, the bacterial concentration observed at 8 days is 345.1 counts. As the total volume of blood is 5000 ml, the resulting bacterial load from the model at 8 days was 0.07 CFU ml^−1^. The bacteria load in the model during the secondary bacteraemia reached the level reported from the experimental study at 9.6 days. The bacteria concentration in the blood at this time was 5513 counts, which is equivalent to 1.1 CFU ml^−1^.

### 3.5. Effect of dose on incubation period

In order to examine the effect of dose on the model output, we altered the value of the initially ingested dose of bacteria such that *B*_0_ was defined as a concentration of 10^3^. From the experimental study, cases with a dose of 10^3^ reported a median incubation time of 9 days to TD (IQR 6.5–13 days); and the quantitative blood culture performed at TD showed median bacterial loads of 0.5 CFU ml^−1^ (IQR 0–1.2).

The output of the mathematical model showed the bacterial concentration at 9 days to be 0.03 CFU ml^−1^ (189 total CFU count); and the bacterial load reached the level observed in the experimental study at 10.5 days, one day longer than the higher dose of 10^4^ (table 2). In both the experimental study and the mathematical model, an increase in ingested dose resulted in an increase in bacterial concentration in the blood by a ratio of about 2.1, leading to a shorter incubation period (table 2).

**Table 2.** Values from mathematical model and experimental study showing the effect of dose on the incubation period.

	experimental study (Waddington *et al*. [40])		mathematical model	
challenge dose and mathematical model initial state	time to typhoid diagnosis (median (IQR))	bacterial count at typhoid diagnosis (median (IQR))	time to reach typhoid diagnosis bacterial levels (0.5 and 1.1 CFU ml^−1^)	bacterial count at the time of typhoid diagnosis (9 and 8 days)
10^3^	9 days (6.5–13)	0.5 CFU ml^−1^ (IQR 0–1.2)	10.5 days	0.03 CFU ml^−1^ (189 total CFU count)
10^4^	8 days (6–9)	1.1 CFU ml^−1^ (IQR 0.4–2.1)	9.6 days	0.07 CFU ml^−1^ (345 total CFU count)

### 3.6. Uncertainty sampling

In the first step of the uncertainty sampling, a hundred random samples were generated for each parameter, and in the second step, a thousand random samples were generated. Electronic supplementary material, appendix S3 shows the probability distribution functions of 1000 randomly generated samples for each parameter displaying the shape of the distribution and the range of the values selected.

### 3.7. Sensitivity analysis

We compared the output of all simulated models and identified six parameters as the most sensitive including: *β*, *α*, *μ*, *C*, *θ* and *τ*_2_. This may be expected from the consideration of the equations themselves as discussed in the model analysis section above as these contribute to the bacterial growth rather than simply tracking the transit of bacteria around body. Of these parameters, *α* is the most uncertain as it is the only parameter without supporting evidence from the literature.

**Figure 4.**
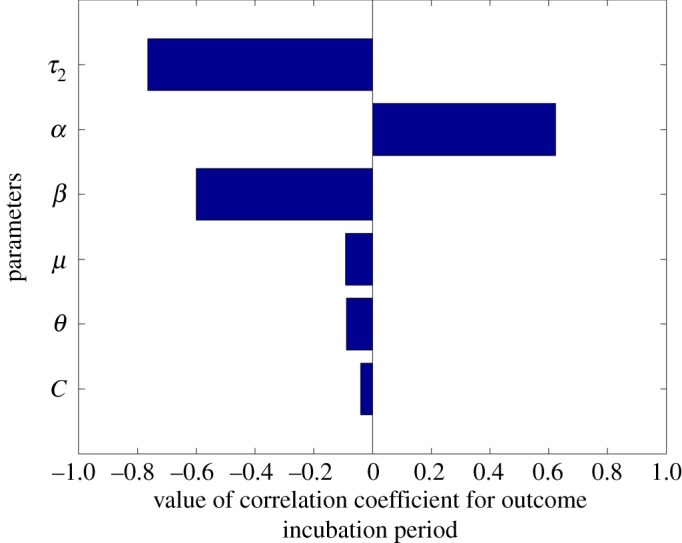
Tornado plot showing the direction and strength of correlation with the incubation period.

**Figure 5.**
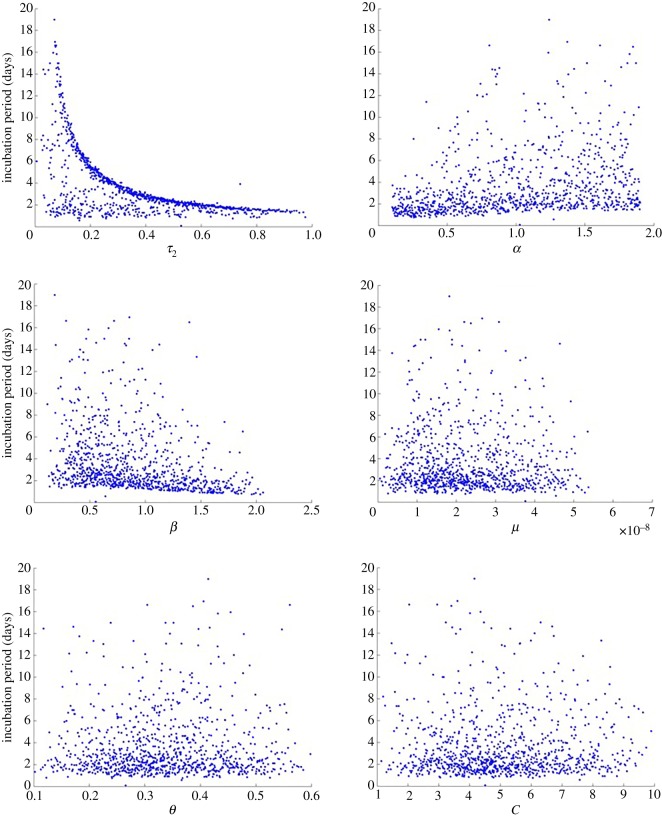
Scatter plots showing correlation with the incubation period.

We ran 1000 reiterations of the model using 1000 randomly generated samples of the six sensitive parameters. Using the output from the simulated models, we conducted a sensitivity analysis by deriving the partial rank correlation coefficients (PRCC) and the corresponding *p*-values (table 3). This provides a measure of the strength of the association between the six sensitive parameters and the incubation period. The parameters with the strongest correlation with the incubation period included *τ*_2_, *α* and *β* with correlation coefficients of −0.7, 0.6 and −0.6, respectively (figure 4). Growth rate in late phase systemic infection (*τ*_2_) was most strongly correlated with the incubation period, where an increase in the growth rate significantly reduced the incubation period (figure 5). With the exception of *C*, all parameters were significantly associated with the incubation period (table 3).

**Table 3.** PRCC of model parameters.

parameter	correlation coefficient	*p*-values
*τ*_2_	−0.77	<0.0001
*α*	0.62	<0.0001
*β*	−0.60	<0.0001
*μ*	−0.09	0.004
*θ*	−0.09	0.005
*C*	−0.04	0.201

## 4. Discussion

We have developed a compartmental model of ordinary differential equations to simulate the infection process of *S*. Typhi in humans in order to estimate the incubation period and identify biological processes that might influence the duration of the incubation period. To the best of our knowledge, this is the first mathematical model on the incubation period of *S*. Typhi in humans.

Gastric acid acts as a barrier to most bacterial organisms including *S*. Typhi [42,43], restricting the proportion of bacteria that continue towards infection. However, its effect can be minimized due to the presence of any condition (drugs or disease) that reduces the stomach acid [43] or the constituents of the ingested food vehicle [44]. Reduced gastric acid may result in shorter incubation period as more bacteria will survive to transit through to cause infection. In our model, we have eliminated the effect of gastric acid allowing nearly all bacteria to exit the stomach, mirroring the experimental study by Waddington *et al.* [41] where sodium bicarbonate was used to restrict the effect of gastric acid.

The bacterial population in the colon is mainly determined by the rate of shedding (*α*) and the replication rate (*β*); hence, the difference of both parameters represents the net growth rate of bacterial population in the colon. If the net population of bacteria is greater than one (*β* > *α*), infection would most likely proceed to the systemic phase as bacteria will be available in the colon to migrate into the caecal lymph node (CLn). The interaction between *α* and *β* means that increasing the value of either parameter produces contrasting effects of equal magnitude on the incubation period, as shown in the tornado plot (figure 4).

The quantitative microbiology of *S*. Typhi in stool is currently unknown; hence, a biologically plausible value of *α* had to be assumed. This introduced some uncertainty into our model, which was further examined in the sensitivity analysis. The output of this analysis indicated that *α* is an important determinant of the incubation period. The value of *β* = 1.8*α* is biologically plausible and was deliberately selected using the Waddington study as a validation dataset independent of the parametrization.

According to our model, bacteria persist in the colon for the duration of the infection process (figure 2) and this is consistent with the literature where shedding has been reported to occur for the duration of infection and even for a few days after antibiotic treatment [45]. In cases without treatment, shedding can occur for up to three months [45,46]. An experimental study showed that participants had positive stool cultures for as long as three weeks and all participants were culture negative six weeks after challenge [47]. In another challenge study, stool cultures were positive from 72 h after ingestion and for the two-week duration of the experiment when samples were collected [41]; however, the proportion of participants shedding [41] and probability of stool shedding [47] declined as the days post challenge increased.

Another critical point for infection, which also represented a major bottle neck, was the migration of bacteria to the CLn (*μ*). At this point, bacteria had a very low probability of migration and the likelihood of infection occurring largely depended on the bacteria crossing this threshold, thus encountering a major bottleneck. This was also observed in a mouse experimental study conducted by Kaiser *et al.* [29]. According to the sensitivity analysis, an increase in the migration rate will reduce the incubation period as the process of systemic invasion is quickened.

The net growth rate of bacteria in the liver and spleen during late phase systemic infection represents the last critical point for infection. Bacterial growth in this phase was strongly correlated with the incubation period and from figure 5, we can deduce that this is a dominant parameter for the distribution of the incubation period. As the growth rate increases, the number of bacteria available to enter into the bloodstream increases, and ultimately, this reduces the incubation period. This conclusion is logical as it is the final opportunity for growth in the last step of the infection process before the onset of the secondary bacteraemia. It is, therefore, plausible for any changes to bacterial growth rate at this point to significantly alter the incubation period.

The concentration of bacteria in the blood during the secondary bacteraemia is predominantly made up of bacteria exiting the spleen and liver; however, according to the sensitivity analysis, altering the exit rate has limited effect on the duration of incubation period. Very early on in the onset of the secondary bacteraemia, the spleen contributed more to the bacterial concentration; however, as infection progressed, the bacterial concentration was sustained by bacteria exiting the liver. This is shown in figure 3 where the bacterial population in the liver was initially higher than the spleen, but subsequently, the bacterial population in the spleen increased and remained consistently higher for the duration of the infection. The identical replication rate (represented by *τ*) in both organs, coupled with the difference in entry and exit rates, with the liver having higher rates, may explain the alteration in bacterial concentration and contribution observed between the liver and spleen.

Comparing the output of our model to data from a human experimental study, the estimated incubation period from our model was 9.6 days. This value was similar to estimates from other experimental and observational studies. A large experimental study conducted over 16 years [48] reported a mean incubation period of 11.4 days with a median of 9 days. Another experimental study reported a shorter incubation period of a median of 6 days (IQR 5.1 to 7.8) in a control group with no vaccination who received a challenge dose of about 10^4^ [49], similar to our initial value. In a systematic review conducted to estimate the distribution of incubation period and identify influencing factors [50], the mean incubation period between subgroups was reported to range from 9.7 to 21.2 days with previously vaccinated cases reporting longer incubation period. In an observational study of a large outbreak associated with Spanish spaghetti, the mean incubation period was 10.5 days with a median of 8 days [51]. In a community outbreak associated with corned beef, the mean incubation period was reported to be 9.1 days with a median of 9 days [9].

According to our model, the bacterial levels in the blood at the time of diagnosis as defined by Waddington *et al.* [41] were 0.07 CFU ml^−1^ which was lower than the 1.1 CFU ml^−1^ they reported, although similar values of low bacterial concentration of about 0.1 CFU ml^−1^ have been detected in the blood of typhoid cases [52,53].

The output of the model has shown that ingested dose, which could be a proxy for attack rate, influences the duration of the incubation period. In both the mathematical model and the experimental study, a 10-fold increase in the ingested dose reduced the incubation period by 1 day. This relationship has also been reported in some observational studies where an increase in the ingested dose shortened the incubation period [7,54].

Although the effect of vaccination has not been considered in this model, vaccination has been reported to prolong the incubation period [50,55]. The effect of vaccination has been studied in an experimental study [49] where the time to microbiological diagnosis was similar in participants with and without vaccination; however, the median time to clinical diagnosis, which also represented the incubation period, was longer in the vaccine groups with reports of 8.5 and 10.4 days compared to the control group reporting 6.8 days. Some vaccines did not appear to affect the shedding pattern or attack rate [47,49]; however, the level of bacteraemia was lowered. In order to achieve this effect, we can speculate that the most likely point of vaccine action will be restricting the replication of bacteria in the systemic organs, thereby reducing the rate of *τ*_2_.

According to Grant *et al.* [18], individual immunological mechanisms can have different progressive effect on the bacterial population. Hence, the immune response in one phase of the infection may be different from another phase of the infection as the bacteria encounter resident phagocytes, polymorphonuclear neutrophils and intracellular control mechanisms during the course of infection. The reduced exit rate of bacteria from the systemic organs to the blood at 1% of the initial entry rate may represent another possible adaptive immune response to slow down the process of infection. At this stage, the bacteria have survived all immunological mechanisms and the onset of sepsis in inevitable; however, slowing down this process might enable further innate responses to intervene or maybe even allow acquired responses to kick in, intercepting the onset of the secondary bacteraemia.

The purpose of our model was to estimate the incubation period of typhoid fever and did not focus on the progress of illness afterwards. Nevertheless, according to our model, the concentration of bacteria in the blood during the secondary bacteraemia continued to increase following the onset of illness at 9 days and this was ongoing even after three weeks of illness. This does not follow the pattern observed in actual human infection where the bacterial counts in the blood decrease with increasing duration of illness [52], such that by the fourth week, the bacterial concentration is 82% less than it was in the first week. Although death typically occurs after the third week of disease, in cases that survive, the fever declines in the fourth week without antibiotic therapy [56].

Our model was a deterministic. Given the unknown shedding rate, we have forced bacterial growth in the colon by making *β* > *α* to ensure that infection progresses to the systemic phase. This, however, could result in unnatural timing given the fractional entry of bacteria into the CLn and so a stochastic model framework might have advantages. Although this is a limitation of our model, it does not undermine the output or the knowledge contributed by this work as it is the first attempt at within-host mathematical modelling of the incubation period of *S*. Typhi in humans. However, further work involving a stochastic model framework is recommended.

Our study showed the application of mathematical models in understanding biological processes and estimating parameters of infectious diseases. The model has been useful in identifying factors intrinsic to the infection process that influence the incubation period and suggests that the late phase net replication in organs and net replication rate in the colon are key determinants of the duration of the incubation period and may explain variation at a population level as much as ingested dose. Other factors, such as vaccination, have not been examined in this study and with the available evidence on the effect of vaccination, further work is required to extend this model to include vaccination.

## Supplementary Material

Model Equations

## Supplementary Material

Growth rate of bacteria in phagocytes

## Supplementary Material

Probability distribution plots

## Data accessibility

No primary data were generated. Data to inform the parameter values were derived from published literature fully referenced to the original accessible source. The code used to develop model simulations is available from the Dryad Digital Repository: https://doi.org/10.5061/dryad.p2gm5q8 [57].
