# Supplementary material for: Within-host mathematical modelling of the incubation period of Salmonella Typhi
Source: R Soc Open Sci. 2019 Sep 11;6(9):182143. doi: 10.1098/rsos.182143 (PMC6774937; doi:10.1098/rsos.182143)
Supplement: Model Equations [file rsos182143supp1.docx]

**Model Equations**

**Invasion of the GI organs**

The rate of migration from the mouth to the colon is defined as:

${\dot{\boldsymbol{B}}}_{\boldsymbol{M}}\boldsymbol{=-\phi}\boldsymbol{B}_{\boldsymbol{M}}$ **(1)**

${\dot{\boldsymbol{B}}}_{\boldsymbol{S}}=$ $\boldsymbol{\phi}\boldsymbol{B}_{\boldsymbol{M}}\boldsymbol{-\delta}\boldsymbol{B}_{\boldsymbol{S}}$ **(2)**

${\dot{\boldsymbol{B}}}_{\boldsymbol{D,1}}\boldsymbol{=\gamma\delta}\boldsymbol{B}_{\boldsymbol{S}}\boldsymbol{-}\boldsymbol{\kappa}_{\boldsymbol{1}}\boldsymbol{B}_{\boldsymbol{D,1}}$ **(3)**

${\dot{\boldsymbol{B}}}_{\boldsymbol{D,i+1}}\boldsymbol{=}\boldsymbol{\kappa}_{\boldsymbol{i}}\boldsymbol{B}_{\boldsymbol{D,i}}\boldsymbol{-}\boldsymbol{\kappa}_{\boldsymbol{i+1}}\boldsymbol{B}_{\boldsymbol{D,i+1}}$ **(4)**

${\dot{\boldsymbol{B}}}_{\boldsymbol{C}}\text{ = }\boldsymbol{\kappa}_{\boldsymbol{7}}\boldsymbol{B}_{\boldsymbol{D,7}}\boldsymbol{-(\mu+\alpha-\beta)}\boldsymbol{B}_{\boldsymbol{C}}$ **(5)**

**Invasion of caecal lymph nodes and phagocytes**

${\dot{\boldsymbol{B}}}_{\boldsymbol{LN}}\boldsymbol{= \mu}\boldsymbol{B}_{\boldsymbol{C}}\boldsymbol{+\nu C}\boldsymbol{P}_{\boldsymbol{C}}\boldsymbol{-(\rho+}\boldsymbol{\eta)B}_{\boldsymbol{LN}}$ **(6)**

${\dot{\boldsymbol{P}}}_{\boldsymbol{C}}\boldsymbol{= \theta\rho}\boldsymbol{B}_{\boldsymbol{LN}}\boldsymbol{- \nu}\boldsymbol{P}_{\boldsymbol{C}}$ **(7)**

Therefore, the approximate population of bacteria available in cecal lymph node and phagocytes is:

$\boldsymbol{T=}\dot{\boldsymbol{B}_{\boldsymbol{LN}}}\boldsymbol{+C}{\dot{\boldsymbol{P}}}_{\boldsymbol{C}}$ **(8)**

**Onset of primary bacteraemia**

The onset of primary bacteraemia and the transfer of bacteria into the organs was described as:

${\dot{\boldsymbol{B}}}_{\boldsymbol{B}\boldsymbol{1}}\boldsymbol{= \eta}\boldsymbol{B}_{\boldsymbol{LN}}\boldsymbol{-}\left( \boldsymbol{\omega+ \sigma} \right)\boldsymbol{B}_{\boldsymbol{B}\boldsymbol{1}}$ **(9)**

**Invasion and replication of bacteria in systemic organs**

Net bacterial growth in systemic infection is defined as:

${\dot{\boldsymbol{B}}}_{\boldsymbol{S}}\boldsymbol{=}\boldsymbol{\omega}\boldsymbol{B}_{\boldsymbol{B}\boldsymbol{1}}\boldsymbol{+}\boldsymbol{\tau B}_{\boldsymbol{S}}\boldsymbol{-}{\boldsymbol{(}\boldsymbol{\Upsilon}\boldsymbol{\omega)B}}_{\boldsymbol{S}}$ **(10a)**

${\dot{\boldsymbol{B}}}_{\boldsymbol{L}}\boldsymbol{=}\boldsymbol{\sigma}\boldsymbol{B}_{\boldsymbol{B}\boldsymbol{1}}\boldsymbol{+\tau}\boldsymbol{B}_{\boldsymbol{L}}\boldsymbol{-(}\boldsymbol{\Upsilon}\boldsymbol{\sigma)}\boldsymbol{B}_{\boldsymbol{L}}$ **(10b)**

**Onset of secondary bacteraemia and clinical symptoms**

${\dot{\boldsymbol{B}}}_{\boldsymbol{B}\boldsymbol{2}}\boldsymbol{= (}\boldsymbol{\Upsilon}\boldsymbol{\omega)}\boldsymbol{B}_{\boldsymbol{S}}\boldsymbol{+ (}\boldsymbol{\Upsilon}\boldsymbol{\sigma)}\boldsymbol{B}_{\boldsymbol{L}}$ **(11)**

where:

$\phi$ is the transit time from mouth to stomach;

$\delta$ is the gastric emptying rate;

$\text{γ}$ denotes the fraction of bacteria that successfully migrate to the duodenum;

$\kappa_{i}=\kappa/7$ where $\kappa$ is the mean transit time through the small intestine and $i=1 ,..,7$ are the 7 compartments in the small intestine reported by Yu;

$\beta$ represents the rate of replication in the colon;

$\alpha$ is the rate of bacteria that is shed from the colon through faeces;

$\mu$ is rate of bacterial migration;

$B_{M}$ is the bacterial population in the mouth;

$B_{S}$ is the bacterial population in the stomach;

$B_{D}$ is the bacterial population in the small intestine;

$B_{C}$ bacterial population size in the colon.

$C$ denotes the number of bacteria in the phagocyte at time of rupture

$P_{C}$ represents the number of invaded phagocytes

$\nu$ represents the rate of phagocyte rupture

$\rho$ denotes the rate of phagocyte invasion

$\theta$ denotes the fraction of bacteria infecting phagocytes

$T$ represents total population of bacteria in both cecal lymph nodes and phagocytes

$\omega\mathrm{and} \sigma$ are transfer rates of bacteria from blood to the spleen and liver

$\Upsilon$ represents the reduction in flow back to the blood

$\tau$ represents the net growth of bacteria in organs during the invasion and replication phases and so $\tau=\tau_{1}$ for early times ($t<t_{1}$) and $\tau=\tau_{2}$ otherwise

$B_{S}and B_{L}$denotes the bacterial population in the spleen and liver
