# Supplementary material for: Within-host mathematical modelling of the incubation period of Salmonella Typhi
Source: R Soc Open Sci. 2019 Sep 11;6(9):182143. doi: 10.1098/rsos.182143 (PMC6774937; doi:10.1098/rsos.182143)
Supplement: Growth rate of bacteria in phagocytes [file rsos182143supp2.pdf]

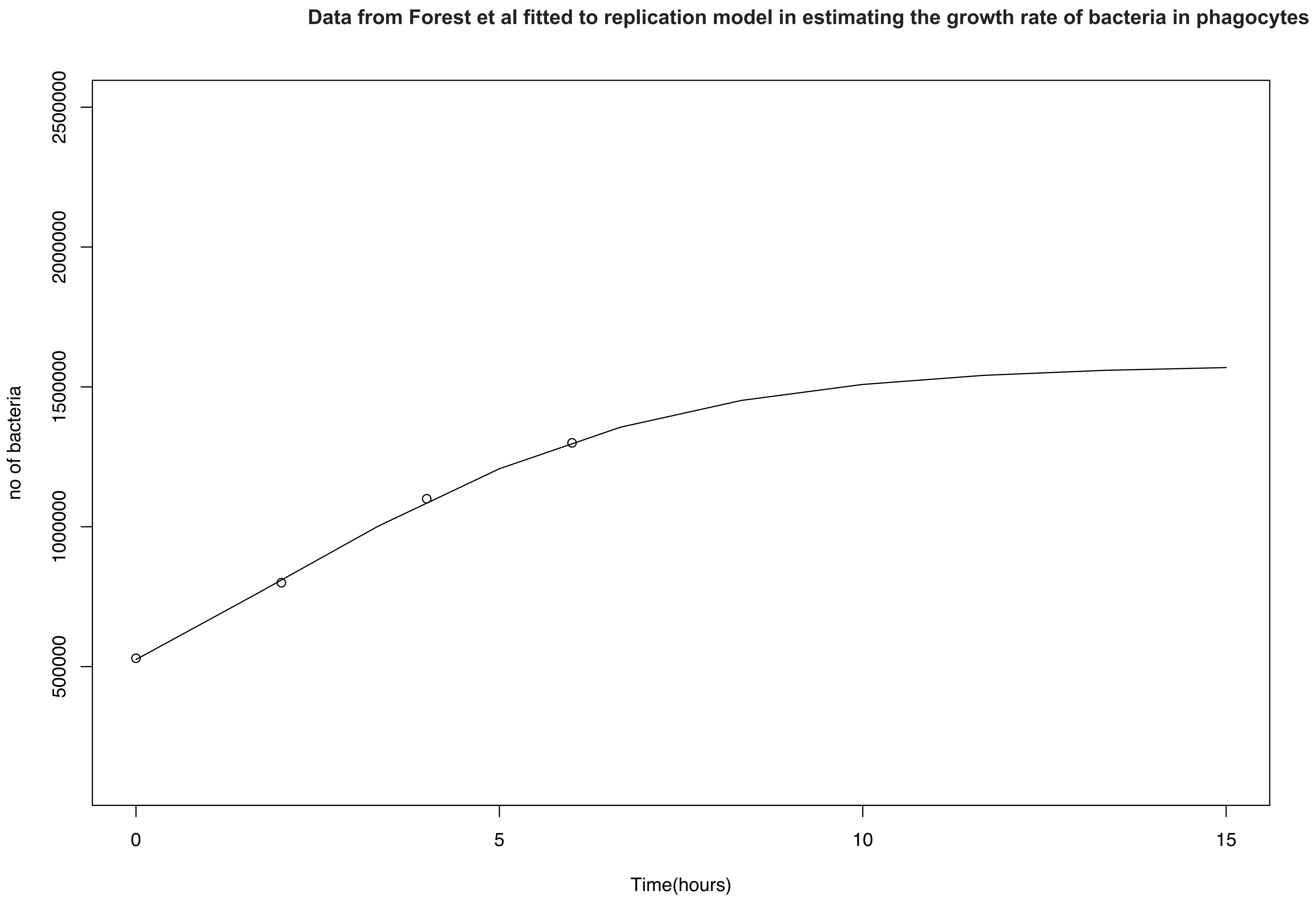
Data from Forest et al fitted to replication model in estimating the growth rate of bacteria in phagocytes
no of bacteria
500000
1000000
1500000
2000000
2500000
0
5
10
15
Time(hours)
