# Supplementary figures and images for: Within-host mathematical modelling of the incubation period of Salmonella Typhi
Source: R Soc Open Sci. 2019 Sep 11;6(9):182143. doi: 10.1098/rsos.182143 (PMC6774937; doi:10.1098/rsos.182143)

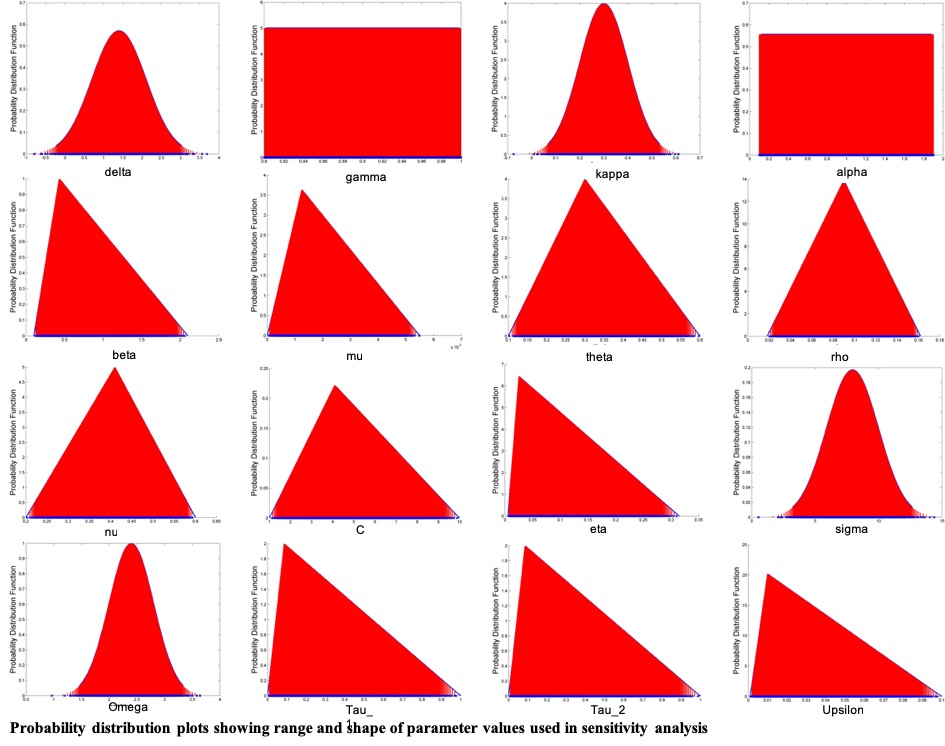

Supplement: Probability distribution plots [file rsos182143supp3.jpg]
